# A Differential Approach to Form and Site of Peptic Ulcer

**DOI:** 10.1038/s41598-019-44893-x

**Published:** 2019-06-18

**Authors:** Walter Rau, Christoph Hohaus, Eike Jessen

**Affiliations:** 1 Department of Surgery, Oberhavel Kliniken, Hennigsdorf, 16761 Germany; 2 The Peoples Friendship University of Russia, Institute of Pathophysiology, Moscow, 117198 Russia; 3 Technical University, Institute of Informatics, Munich, 81333 Germany

**Keywords:** Peptic ulcers, Pathogenesis, Biological physics

## Abstract

The structural organization of intestinal blood flow is such as to allow for intramural collateral flow. Redistribution phenomena due to different local metabolic demands may lead to an impaired perfusion of parts of the intestinal wall which will display a characteristic pattern. Based on Ohm’s and Kirchhoff’s laws, a differential analysis of the gastric vascular bed bridges the gap between basic physiological concepts and traditional anatomical, pathological and clinical knowledge. An ulcer of the intestinal wall becomes understandable as a non-occlusive infarct based on a supply/demand conflict in an anisotropic structure as it can be found in the upper and lower gastrointestinal tract of man.

## Introduction

“*Such a kind of attempted explanation should have to take into consideration not only the initial formation or the later chronic condition of the ulcer*, *but at the same time its site and form*”. LUDWIG ASCHOFF^1^ p. 494 (translated by the author from the German original).

One of the oldest problems of pathology is understanding how an ulcer, a localised and circumscribed necrosis of the intestinal wall, extending below the level of the muscularis mucosae, may occur in such a well perfused structure as the human stomach or duodenum. Historically, the multitude of theories on the nature of peptic ulcer was grouped into two families: the effect of harmful agents, like hydrochloric acid, working from the mucosal surface versus an underlying disturbance of gastric or duodenal blood flow, to explain the obvious pathological picture of a circumscribed lesion displaying ischaemic necrosis. With the aetiology of hypersecretion well understood^2,3^, it remains completely unclear how an ubiquitous intraluminal field-factor, such as hydrochloric acid, could nevertheless produce a discrete and limited loss of tissue substance. Also a surface phenomenon as a generalized gastritis has never been able to correct this shortcoming. The alternative explanation of ulcer formation by classical vascular theories repeatedly failed on account of the rich arterial supply of the organ. Although Virchow’s proposal of an infarctive pathogenesis has never been disproven, the affected vessel he proposed as the most probable cause for a haemorrhagic necrosis could never be demonstrated^4–6^. Rather than looking for deviant forms of an infarct we got used to discuss ulcer expression as an imbalance of aggressive and defensive forces, without understanding how the latter causally depend on the former. To better understand the localised nature of full-walled necrosis of the intestinal wall and the singularity of the phenomenon, we will have to understand how these two factors depend on each other^7^.

**Figure 1:**
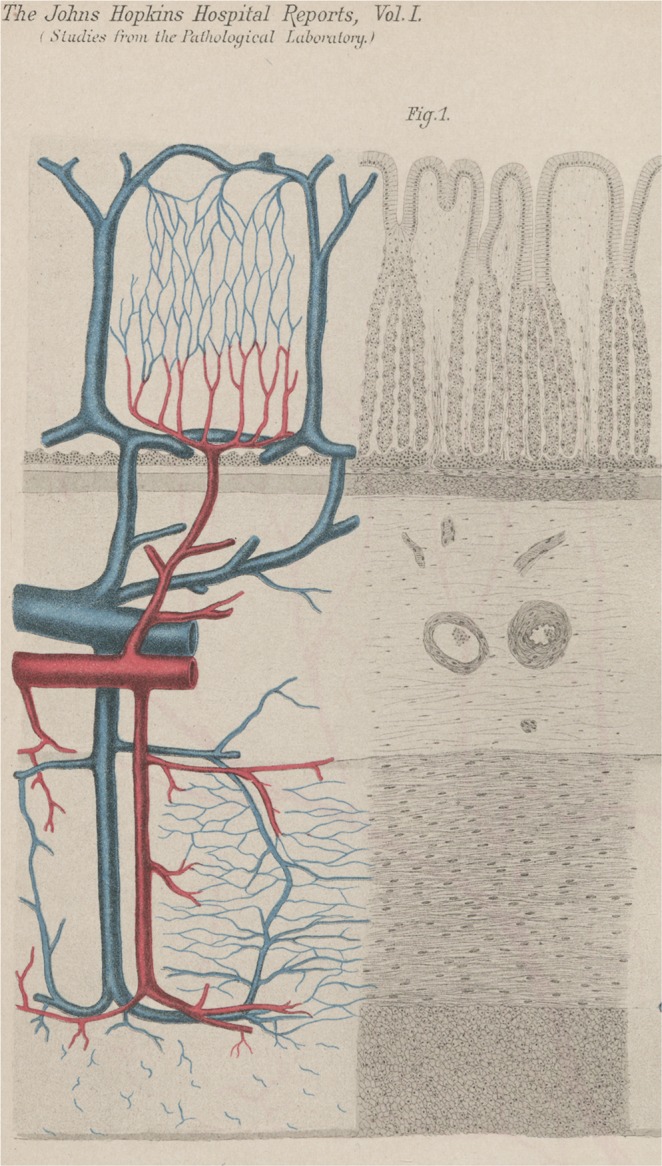
Basic vertical arterial element of the gastric wall. Original Figure from F. Mall^35^, which in its left part shows a 3D-reconstruction of the vessels from serial sections of the stomach-wall. The submucous plexus is the central offspring of the arteries supplying the mucosa and also a varying part of the muscle layer. Every decline in pressure in the submucous arterial plexus will lead to a reduction in flow in the dependent parts of the gastric wall. Detail of Plate III, Fig. 1 from Johns Hopkins Hospital Reports 1^35^. By Courtesy of Johns Hopkins University Press. This figure is not covered by the CC BY licence. Credits to ©Universitätsbibliothek der Humboldt-Universität zu Berlin, Historische Sammlungen: Ia 40920:F4. All rights reserved, used with permission.

The stomach exhibits the rich arterial perfusion of an upper abdominal secretory organ. As in other hollow organs its walls are typically not supplied by extramural end-arteries. Although such vessels have been described^8^ their proposed occlusion does not explain the morphology of a gastric or duodenal ulcer. Neither does their distribution match the monotonous pattern of ulcer localisation, nor does their proposed occlusion explain the characteristic form of the lesion. In the human stomach the main arterial blood supply is located in-between the mucosa and the muscle mantle. From this central location a continuous arterial plexus provides blood flow to both layers, giving off branches to the mucosa and the muscle mantle as well. This submucous plexus provides arteries of 200 *μ*m to 1000 *μ*m in diameter^9^, which supply the terminal vascular bed of the metabolically active mucosa as well as in a retrograde fashion a varying part of the surrounding muscle layer. The arteries of this internal collateral system are often larger in diameter than their muscle-perforating external sources. The plexus is capable of supplying each and every point of the gastric wall. This internal arterial supply is independent of local contractions of the muscle mantle, which constitute a varying extravascular resistance to muscle-perforating arteries. We are not looking for the effect of single occluded vessels but for the performance of the complex arterial network as a whole (Fig. 1).

During a gastroscopy the endoscopist will never see a pale strip of mucosa moving downwards with peristaltic contractions of the associated muscle mantle. This is avoided by the collateral competence of the submucous plexus, a factor which impacts upon everyday surgical practice. Thus, extramural arteries can be divided rather freely without producing a necrosis of the gastric wall. For example, if the stomach is used for oesophageal replacement three out of four of its principal arteries are divided without producing a necrosis of the dependent part. This can be safely used for a replacement of the tubular oesophagus as long as the development of wall stress in the stomach wall is reliably avoided and the submucous plexus stays intact. In animal models only the ligation of arteries at the level of the submucous plexus itself is able to produce typical ulcers of the gastric wall^10^.

**Figure 2:**
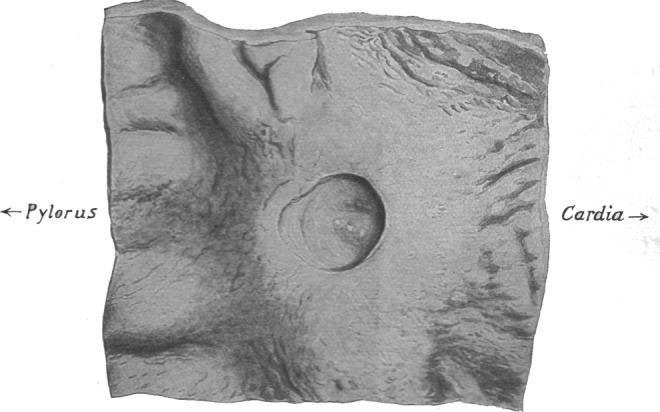
Morphology of a gastric ulcer in the antrum. L. Aschoff described the form of gastric ulcer as *Reibungsform*, assuming frictional forces to secondarily deform the ulcer. Ischaemic necrosis due to an occluded vessel in an end-artery model should typically produce cylindrical ulcers of the gastric wall. Aschoff found that, in reality, gastric ulcers express a steep edge cranially and run out shallowly distally - often producing the form of a staircase. Reproduction of Fig. 515; p. 760 from Aschoff^11^.

Aschoff observed that gastric ulcers do not show the axial symmetry of a cylindric loss of tissue substance, which would be expected if they were due to to a single occluded vessel. Instead, they show the asymmetrical morphology of a staircase with the axis of the ulcer pointing away from the parietal cell mass towards the pylorus (Fig. 2)^1,11^. Aschoff tried to explain this by propulsive peristaltic forces in the course of digestion. He assumed these forces to be responsible for the secondary development of the tilted funnel, which he described as “*Reibungsform*” (frictional form). However, the form of gastric ulcers which he described is not a secondary characteristic of chronic ulcers alone - acute lesions also exhibit the same feature as Hauser noticed soon after Aschoff’s attempted explanation^12^. When viewing a gastric ulcer the naïve examiner has the impression of an eccentric target lesion, with the extent of the damage declining distally. But the rings of the cockade do not share a common centre, where the injury is supposed to be maximal, but instead share a common edge, represented by the mucosal boundary between antrum and corpus.

The vast majority of gastric ulcers is situated in the gastric antrum. Their position seems to vary with the patient’s age^13^. Circumferentially, the ulcer is generally limited to the lesser curvature of the stomach^14,15^. The longitudinal position of gastric ulcers tends to shift to a more proximal location with increasing age. This correlates with an involution of the highly specialised acid-secreting mucosa in elderly persons^16^. Oi had shown in 1959 that gastric ulcers are located close to the mucosal boundary, between the acid-secreting corpus and the neighbouring antrum, always developing at the antral side of the mucosal margin^17^. Out of 499 observations of gastric ulcers only 21 were found more than 2 *cm* away from the histological border, and 3 of them inside the acid-secreting mucosa itself. By directly staining the parietal cells, he could show later that 2 of these ulcers displayed a dystopia comprising a rest of antral mucosa at their margin^18^. A correlation of at least 95% is uncommon for any biological phenomenon. It points to the possibility of a causal relationship between the localisation and the nature of the lesion which we have yet to demonstrate^15,19^.

Although both the form and site of human gastric ulcers have been documented in depth, Aschoff’s postulate constitutes a timeless challenge to every formal theory of ulcer formation. On no other surface of the human body does a circumscribed loss of tissue substance with defined margins occur without the loss of local oxygen supply. In every vertebrate organism the transport of oxygen over longer distances is strictly confined to arteries. It is therefore highly relevant to review the anatomy of the arterial network as the basis for the structural details of any limitation of arterial blood flow even in such a complex organ as the stomach. Such functional disorders of local perfusion, based on a reversal of arterial flow, are well known in simpler networks, of which the subclavian steal phenomenon is the earliest and best described. Mathematics presents the only formal way to deal with the functional consequences of a complex arterial haemodynamic network in man and to explore the patterns of such phenomena. Using an analysis of the gastro-duodenal vascular bed in man, as applied to the two-dimensional submucous arterial network, we provide an explanation of the singularity of the resulting necrosis and are able to compare the morphology of the expected dissipative pattern of local blood flow to the well described form and site of the human ulcer.

## Theoretical Background and Development of the Model

### Discrete haemodynamic model

**Figure 3:**
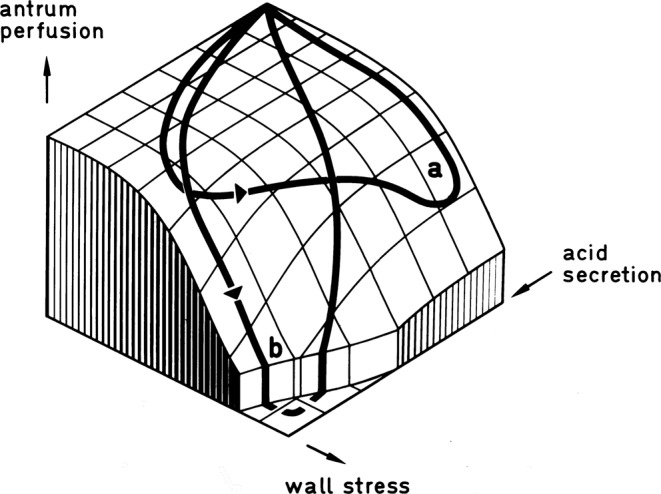
Theoretical prediction of the conditions of a submucous steal-phenomenon in a discrete three-compartment model of the stomach. Every possible value of MBF in the antral borderline segment is part of the computed surface. A decline of MBF below the minimum perfusion pressure is physiologically avoided by a sequential occurrence of acid-secretion and wall-stress. (a) In a single cavity stomach gastric emptying takes place after the end of the secretory phase. This can be overridden experimentally by stimulating acid secretion in the gastric emptying phase. (b) Such an imbalance of secretion and motor activity is also typical for gastric ulcer patients. Figure from^19^, where the discrete haemodynamic model is discussed in detail.

Using a haemodynamic three-compartment model of the stomach we had shown earlier that any decline of local vascular resistance in the acid-secreting area should lead to a fall of mucosal blood flow [MBF] in the adjacent antral segment, when limited influx conditions apply^19^. Such a supply/demand conflict is well in-line with experimental findings on MBF, when acid secretion is stimulated during the gastric emptying phase. Experimentally such a redistribution of flow in larger arteries of the stomach wall was first described by Rene Menguy in^20^. It has been criticised as based on methodical errors by others who could not reproduce his findings using a different method^21^. The discrete haemodynamic model predicted that such a redistribution of flow will only take place when the stomach is not empty and a positive intragastric pressure coincides with acid secretion (Fig. 3). Experimentally Menguy’s findings are reproducible by stimulating acid secretion in the postprandial phase. Under these conditions the theoretically predicted submucous steal phenomenon^19^, a redistribution of flow from the antrum to the corpus, could be detected using 9 *μ*-microspheres in dogs^22^ and iodo-^14^C-antipyrine-autoradiography in ferrets^23^. While in dogs terminal MBF declined in the whole of the antrum, the effect in the ferret was spatially limited to the mucosal boundary. The submucous arterial plexus is detectable as a structural detail in every vertebrate species, but considerable differences between species exist in the width of its meshes^24^. For man, the geometry of these meshes, which are spread out in two dimensions as an arterial network between the muscular - and the mucosal layer, was described first by Duvernoi as early as in^25^.

### Cellular automaton

**Figure 4:**
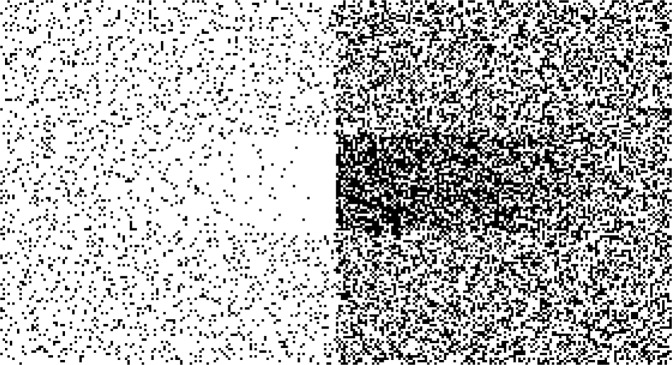
Output of the cellular automaton as given in^26^. On the right side the corpus mucosa shows a higher metabolic demand compared to the antral side (left). In the centre the course of the submucosal arteries is AXIAL while at the upper and lower margin it is assumed CIRCULAR. In the human stomach AXIAL represents the typical pattern of submucosal arteries at the lesser curvature. Here submucosal arteries are running perpendicular to the mucosal boundary. The result of the simulation shows the expected distribution of blood flow under limited inflow conditions. The left part of the pattern resembles Aschoff’s *Reibungsform* of gastric ulcer. For details of the model and the underlying program-code see^26^.

While the location of gastric borderline lesions has been described by the discrete model mentioned above the resulting pattern of blood flow at the boundaries of the acid-secreting mucosa has been simulated earlier by use of a simple cellular automaton^26^. The model took into account the different metabolic demands of adjacent mucosae (a dimensionless variable varying from 10 to 100), limited influx conditions as a Boolean variable and the preferential direction of submucous arteries (AXIAL or CIRCULAR). The cellular simulation produced patterns which matched the form and localisation of gastric ulcers in man^11,15,17^. In particular the model showed that, even under reduced influx conditions, such a pattern will only develop when the adjacent mucosal types have a sufficient arterial connection at the submucous plane (Fig. 4). This is the case when the preferred direction of larger arteries in this structure is AXIAL, perpendicular to the mucosal boundary. The model considered the submucous plexus as the arterial source for mucosa and muscularis propria as well. When such a fluctuation lasts long enough, it will in the end necessarily lead to a functional infarct of the gastric wall, the form of which reflects the simulated pattern of flow. The phenomenology of duodenal ulcers however cannot be understood by acid secretion and wall stress alone. To understand these findings and to demonstrate the influence of an additional variable such as the presence of unbuffered intraluminal acid on the developing pattern in the duodenal wall, we will consider a differential model of intestinal blood flow.

### The differential hemodynamic model of intestinal blood flow

**Figure 5:**
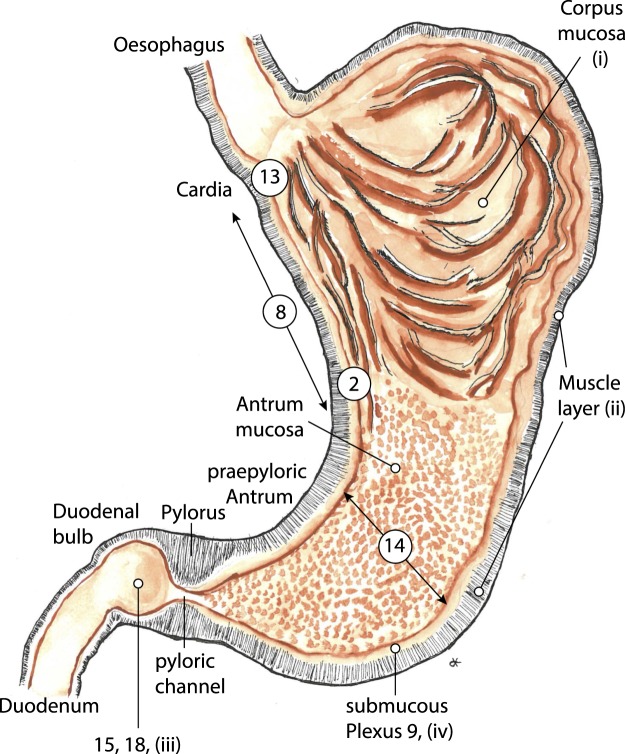
Sketch of the anatomical regions of the foregut and the adjoining duodenum as referred to in the text. Viewed from the luminal side these regions differ in their histological lining. The oesophagus is lined with a non secreting squamous epithelium; the metabolically highly active corpus mucosa contains acid-producing parietal cells whilst the mucosa of the antrum generally does not. The border to the duodenal mucosa is generally found over the pylorus. The muscle layer is most pronounced in the gastric antrum with the pylorus muscle being its boldest structure. The external vascular supply of the foregut, which is not illustrated here, is granted by arteries which enter at both curvatures (Fig. 6). The Arabic figures give the localization of the corresponding figures, lowercase Latin figures give the 3D location of the variables considered. Figures 15 and 18 are located on the anterior surface which has been dissected away.

For the deductive attempt of a differential haemodynamic simulation we will have to perform a rather extreme model reduction. Taking the well-described anisotropic structure of the human submucous arterial plexus as a morphological variable (iv) we will have to define the required physiological variables (i–iii) first (Fig. 5).

#### (i) Secretion of hydrochloric acid and local blood flow

The energy requirements of acid secretion can be estimated from the pH gradient between the intravascular (pH = 7.4) and the intraluminal (pH = 1) compartments. The change in free energy for the secretion of 1 Mol of *H*^+^ into the gastric lumen can be calculated as:

1 $${\rm{\Delta }}G=RTln\frac{{10}^{-1}}{{10}^{-7.4}}\approx \mathrm{38[}kJ/Mol]$$

Acid secretion is accomplished by the *H*^+^*K*^+^-ATPase, which is found exclusively in the parietal cells. These acid-secreting cells are located in the basal aspects of the corpus glands. This region of the corpus mucosa is supplied by an independent arterial network woven around these parts of the gastric glands^27^. To provide the energy required, arterial blood flow in the terminal vascular bed of the acid-secreting mucosa is adapted on a local level. Acid secretion and local mucosal blood flow (MBF) in the acid-secreting area are closely correlated.

In man such a simultaneous rise of MBF and the onset of acid secretion has been confirmed by direct observation before the chemical composition of the “alimentary solvent” had been identified. It was first described by the American Surgeon William Beaumont:

“*When empty, the rugae appear irregularly folded upon each other, almost in a quiescent state, of a pale pink colour, with the surface merely lubricated with mucus. On the application of aliment, the action of the vessels is increased; the colour brightened; and the vermicular motions excited. The small gastric papillae begin to discharge a clear, transparent fluid, (the alimentary solvent,) which continues abundantly to accumulate, as aliment is received for digestion*”^28^.

Experimentally, such a correlation of the secretion of acid and MBF has repeatedly been demonstrated in various species using different methods. This is a strictly local phenomenon - such an augmentation of flow with the onset of acid-secretion does not occur in the neighbouring areas of the antral mucosa^21,22,29,30^.

We will later model this behaviour of the terminal vascular bed of the mucosa by varying its haemodynamic resistance. For this purpose, we will focus on local tissue conductivity to arterial blood flow through a given spatial segment, rather than considering the resistance of single vessels. A detailed knowledge of the mechanisms involved in local regulation of blood flow to adapt to local metabolic demand^31^ will not be required to run a simulation of the morphological outcome as a macroscopic phenomenon. Any augmentation of acid secretion will be modelled as a rise in local tissue conductivity *ω*_(*x*,*y*)_ in the acid producing mucosa. Considering a given perfusion pressure this will provide the necessary rise in local MBF. Such a rise in local perfusion of the acid-secreting area should primarily not have any effect on mucosal conductivity in other parts of the stomach, which will be treated as passive elements.

#### (ii) Intraluminal pressure, wall stress and local blood flow

The human stomach not only provides an acid-secreting mucosa in the corpus, it also serves as a receptacle for food with the antrum acting as an organ of trituration. In the single cavity stomach of man, secretion and motor activity are physiologically consecutive. During gastric emptying the stomach develops positive intraluminal pressures by contractions of the surrounding muscle mantle. Arterial influx to the metabolically active mucosa must enter via the muscle-perforating vessels of the muscularis propria and will be limited by any rise of intraluminal pressure. Any such augmentation in wall stress will necessarily lead to a fall in transmural conductivity *α*_(*x*,*y*)_ and may lead to a decline of perfusion pressure in the distributive network further downstream. In man such an effect of intraluminal pressure on intestinal blood flow is directly evident surgically. The effect has also been reproduced and measured in animal preparations. In experimental setups such a negative correlation of intraluminal pressure and blood flow has early been shown to exist for various parts of the intestine^32,33^ and the stomach^34^ as well.

**Figure 6:**
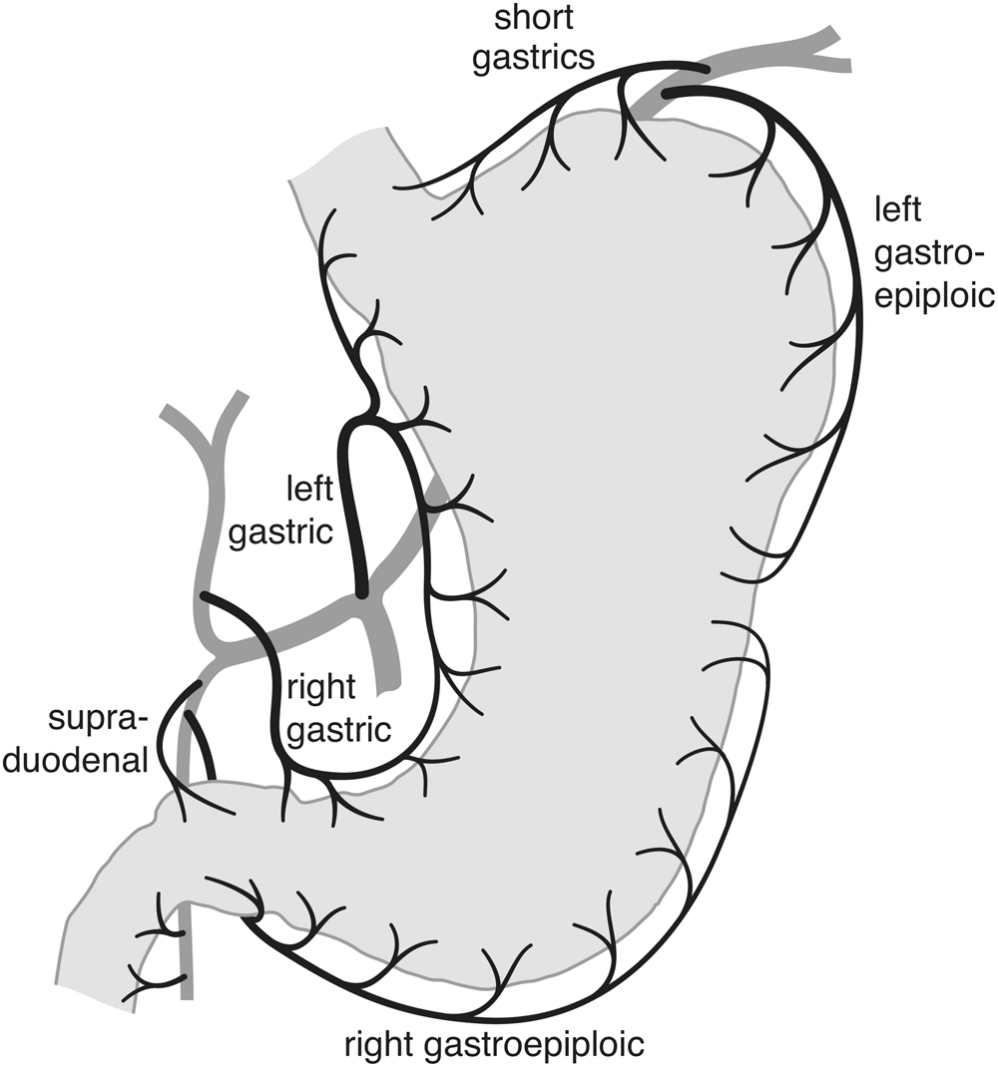
External arterial sources of the foregut. These vessels provide the systemic arterial pressure to the stomach and its neighbouring structures. Before they enter the muscle layer they form a dorsal mesentery (the gastroepiploic arc) at the greater curvature and a ventral mesentery (the gastric arc) at the lesser curvature. While the dorsal blood supply continues throughout the gut, the ventral mesentery ends with the supraduodenal artery^79^ to the duodenal bulb. Peptic ulcer is strictly limited to the foregut which is characterized by a dual mesenteric supply.

A variation of influx to the central arterial network of the submucosa does not solely depend on wall-stress or overall intraluminal pressure which are collinear variables (Laplace’s law). The external arterial influx to the network is not evenly distributed. The local conductivity of muscle-piercing arteries can also be used to model the influence of the anatomical distribution of muscle-perforating arteries itself. In man the stomach and the proximal duodenum show both a dorsal and a ventral mesentery. Supplying vessels enter at both curvatures (Fig. 6). This uneven distribution may have an impact on the pattern of local blood flow distribution. Classic vascular theories have focused on the occlusion of single arteries. The continuous model will show that, even in a complex network, conditions exist which will lead to a conflict between the metabolic demand of the mucous membrane and a limitation in arterial influx to the network itself that will produce characteristic patterns of terminal blood flow to the corresponding layers of mucosa and muscle.

#### (iii) Effect of unbuffered HCl on duodenal blood flow

As a third variable we will have to consider the presence of a long-lasting acid hypersecretion under fasted conditions, by whatever means this is brought about. The presence of unbuffered intraluminal acid will be shown to be a redundant variable for the development of gastric ulcers. To the historic view this may seem somewhat surprising. But unlike the metabolic demand of acid secretion (i) it will not be needed to simulate the asymmetric form and preferred site of gastric ulcer. These sites of necrosis are situated at mucosal boundaries of the acid-secreting mucosa^17^ and prefer Waldeyer’s *Magenstrasse*, the inner aspect of the stomach which extends along the lesser curvature from the cardia to the pylorus^13,14,18^. Taking into account the fixed morphology of the submucous arterial plexus (iv), local disturbances of blood flow in the stomach which show the typical asymmetrical *Reibungsform* of gastric ulcer can, as such, be modelled by variables (i) and (ii) alone.

**Figure 7:**
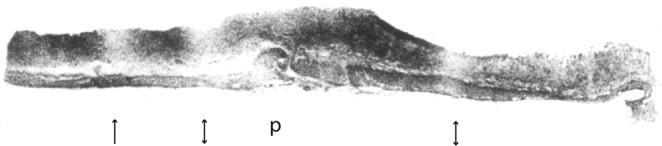
Transmural blood flow in the ferret using iodo-^14^C-antipyrine-autoradiography. The highest levels of MBF will be found in the duodenal mucosa in fasted animals under secretory conditions. The local acid load will produce a rise in local MBF of 400 ml/100 g/min up to 600 ml/100 g/min, which by far exceeds MBF in the acid-secreting gastric mucosa^23^. The autoradiograph shows a longitudinal section, which is taken from the duodenal roof (*left*), including the pyloric muscle (p and the prepyloric antrum. The image, which has been accidentally recorded at the end of the phase of constant arterial invasion of the tracer, contains a further information. With exceedingly high flow rates we can also see an uneven distribution of flow at that point in time. We have interpreted this as a beginning washout effect. It may give a direct hint to the distribution pattern of arterial vasa recta, which originate directly from the submucous plexus. One of them seems to supply only the mucosa (↑) and two of them in a retrograde fashion the muscle mantle as well (↕). Livingstone has recorded the same pattern in a different species^80^. The underlying arterial structure has first been reconstructed by Franklin Mall from serial sections of the dog’s stomach^35^. His original drawing is quoted in Fig. 1.

The intraluminal exposure of the mucosa to free HCl will, however, prove critical to the understanding of duodenal ulcer. Contact of the duodenal mucosa to unbuffered 1-N-HCl will lead to an exceptional rise in MBF, which far exceeds MBF in the acid-secreting areas (Fig. 7). We will show that, in an anisotropic network, this cannot occur homogeneously, even when the mucosal conductivity shows a uniform response. This third variable will later be necessary to understand the site, the axial symmetry and the self-limiting character of deeper duodenal lesions.

#### (iv) Morphology of the submucous plexus in man

A submucous arterial plexus made up of arteries of 200 *μ*m to 1000 *μ*m in diameter is fed by muscle perforating arteries. This plexus is the source of the arteries supplying the mucosa and the inner aspects of the muscularis propria as well. It is important to note that arteries of this magnitude are not resistance vessels but are built to provide an arterial perfusion pressure. Arteries of the magnitude considered here are passive elements which allow blood flow in any direction dictated by local differences in perfusion pressure. Such an arterial plexus can be found in any vertebrate hollow organ, between the outer muscle layer and the metabolically active mucosa. Its varying dimensions reflect the depth of the muscle mantle. In the human stomach the plexus serves as the key arterial supply, not only to the mucosal layer but also in a retrograde fashion to a varying part of the muscularis propria itself. It thus minimizes the effect of local muscular contractions on local blood flow to the terminal vascular bed of the mucosa as well as the muscle-layer (Fig. 1). Its structure has been extensively described in its three dimensional aspects in both the dog^35^ and in man^9,36,37^.

The two-dimensional anatomy of this structure was also the focus of scientific interest in the 1920s^38–41^. The observations at that time focused on peculiarities in the submucosal distribution of vessels at the lesser curvature of the stomach, the prevalent site of gastric ulcer. It has, however, never been possible to find any deficit in the collateral capacity of the submucous plexus to this region. On the contrary, any attempt to deprive the stomach of its main blood-supply by surgically dividing its principal arteries resulted in only a limited necrosis of the parietal parts of the fundus, if at all. The lesser curvature, well collateralised to the oesophagus and the duodenum at the submucous level, always remained viable^42^. Furthermore, the submucosa underneath acute stress lesions does not display any shortage of vessels but an abundance of collateral arteries therein^43^.

**Figure 8:**
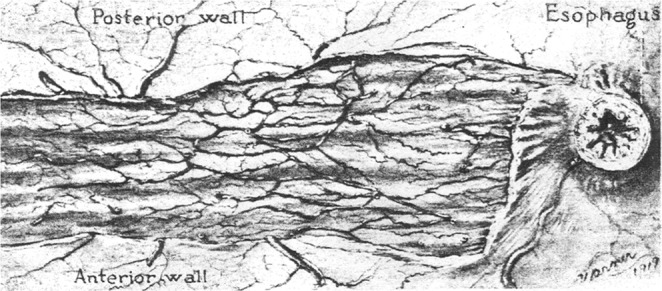
“Dissection illustrating the submucous plexus of arteries on lesser curvature of the stomach. Note their length, size and general direction”. Caption and original illustration (page 380, Fig. 5) from T.B. Reeves^38^.

T.B. Reeves first described the vascular characteristics of this region (Fig. 8):

“*Since the submucous plexus on the lesser curvature is different from that in other parts of the stomach, I shall describe it separately; it is made up by small perforating branches from the main trunks along the lesser curvature. On entering the submucosa these vessels bifurcate and run more or less parallel to each other between the oesophageal opening and the pylorus. They are much smaller, make fewer anastomoses and run more than twice the distance of the same sized vessel in any other part of the stomach*”^38^.

With the intake of food the parietal walls of the stomach will greatly expand and the circumferential direction of submucous arteries therein will become even more pronounced. The lesser curvature and the distal antrum, however, remain inherently stable, leaving the geometry of the submucous network intact. Here, the course of submucous arteries does not show the marked anisotropy around the circumference as it does further up the antrum and in the proximal part of the stomach. In the distal antrum the meshwork of the submucosal plexus shows a homogeneous pattern all around the circumference^37,41^.

This difference in pattern goes back to the embryological development of the stomach. In a 7 mm embryo the organ looks like a “fusiform dilatation” of the foregut. Later growth processes will lead to a change in the shape of the organ. In a 15 mm embryo the final form of the stomach is approached^44^. Before the 12th week, vessels running axially represent the typical finding around the circumference of the stomach. Around the 18th week the arteries of the newly developed parietal walls will show the typical circumferential aspect of submucosal arteries and mucosal plicae similar to those found in the parietal walls of the adult individual^45^.

We will use this given morphological distribution to model the potential conflict between arterial influx and metabolic need. The design of the vascular organization provides us with a unique opportunity to simulate the effect of the morphology of a two-dimensional manifold on the development of a three-dimensional pattern expressed in the gastrointestinal wall. The anatomically defined patterns of submucous arteries will be used in the simulation as morphological constants which only depend on their localisation, direction and calibre. This will then allow us to predict the consequences of the submucous vascular topography on form and site of localised patterns of gastroduodenal blood flow deficiencies.

**Figure 9:**
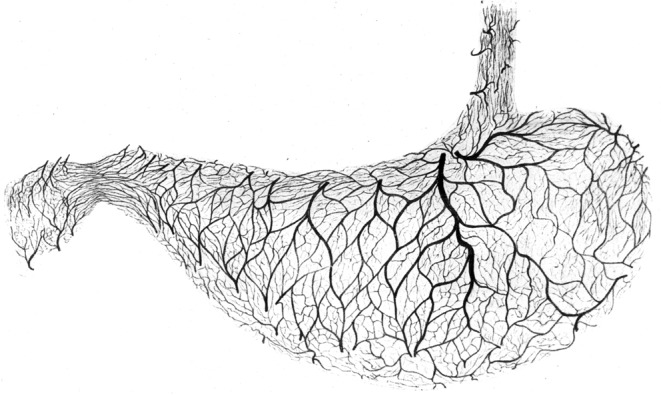
Topographical anatomy of the arterial submucous plexus in man as drawn by Frans Djørup^41^. His original figure (Abb. 15, p. 321) shows the axial course of the submucosal arteries in the oesophagus (*right*), extending into the proximal part of the lesser curvature (*top*). Only on the parietal walls of the stomach does a circular pattern prevail. In the distal antrum such marked differences in pattern around the circumference are not observed - the meshes of the submucous plexus show a more “star-shaped”^37^ pattern in the distal antrum and display a parallel aspect once the pyloric channel is entered (*left*). The anisotropy will become even more obvious when the stomach is filled with food. While the parietal walls will greatly expand, the lesser curvature and the pyloric channel will remain inherently stable. Reprinted by permission from Springer Nature: Zeitschr. f. d. ges. Anat. I. Abt. “Untersuchungen über die feinere topographische Verteilung der Arterien in den verschiedenen Schichten des menschlichen Magens”, Frans Djørup, ©1922. This third party figure is excluded from the open access license.

In the foregut, where a double mesentery outlasts the embryologic development, an axial course of the submucous arteries is found in the oesophagus, the lesser curvature of the stomach and the pyloric channel. In the proximal duodenum it changes to a crisscross pattern between the mesenteric intakes (Fig. 9). Further downstream, a much simpler design is expressed: at the end of the foregut the pattern changes abruptly when arteries from the (only persisting) dorsal mesentery perforate and run circularly around the gut to form an anastomosis on the contra-lateral side. This monotonous pattern of submucosal supply prevails throughout the complete small bowel and the colon down to the lower rectum, where, again, we find a dual mesenteric supply from both sides. The formation of singular ulcers as a solitary phenomenon is strictly limited to those parts of the gut which show a complex bilateral arterial supply (Fig. 6).

**Figure 10:**
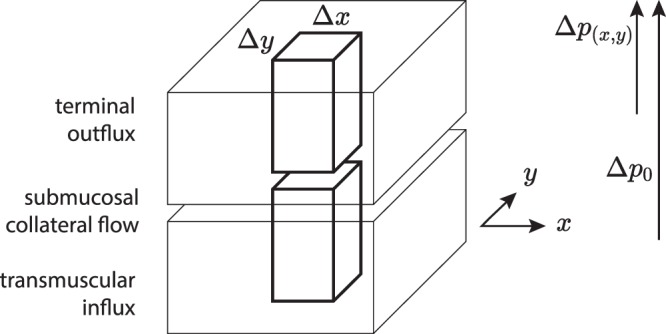
Model of the intestinal wall to derive the differential equation of intestinal blood flow. The model considers an element Δ*x*Δ*y* of the intestinal wall, consisting of a transmuscular influx (*bottom*), a submucosal (*middle*) and a terminal outflow compartment (*top*). This outflow compartment consists of a mucosal component and a part component of the muscle layer, which is supplied by retrograde arteries as well. The systemic arterial perfusion-pressure Δ*p*_0_ reduces over the intestinal wall as a whole. At the submucosal level the variable gradient Δ*p*_(*x*,*y*)_ will serve for the perfusion of the terminal vascular bed. Transmuscular influx *i*_*α*_, terminal outflow *i*_*ω*_ and lateral flow *i*_*λ*_ in the submucosal compartment will be considered.

Such a continuous haemodynamic model will also allow us to study patterns of blood flow away from mucosal boundaries. Originally developed to study redistribution patterns in the stomach^46^, we will use it here as an universal tool to describe blood flow in other regions of the intestine which share the same principal organization (Fig. 10).

### The partial differential equation of intestinal blood flow

Arterial influx Δ*i*_*α*_, outflow Δ*i*_*ω*_ and lateral flow Δ*i*_*λ*_ take place through the intestinal wall as a whole. Storage capacity of the intestinal wall is small so only stationary flow has to be considered.

Arterial influx Δ*i*_*α*(*x*,*y*)_ through the element Δ*x*Δ*y* may be formulated as:

2 $${\rm{\Delta }}{i}_{\alpha (x,y)}={j}_{\alpha (x,y)}{\rm{\Delta }}x{\rm{\Delta }}y$$

(with density of flow = *j*_(*x*,*y*)_). The outflow *i*_*ω*(*x*,*y*)_ through the mucosa (and the retrograde supplied part of the muscle layer) of this element is given by:

3 $${\rm{\Delta }}{i}_{\omega (x,y)}={j}_{\omega (x,y)}{\rm{\Delta }}x{\rm{\Delta }}y$$

Both depend on the gradient of pressure Δ*p*_(*x*,*y*)_ from the submucous plane to the terminal vascular bed and also on the conductivity in the transmuscular influx compartment *α*_(*x*,*y*)_ and the terminal outflow compartment *ω*_(*x*,*y*)_ respectively.

4 $${\rm{\Delta }}{i}_{\alpha (x,y)}={\alpha }_{(x,y)}({\rm{\Delta }}{p}_{0}-{\rm{\Delta }}{p}_{(x,y)}){\rm{\Delta }}x{\rm{\Delta }}y$$

and

5 $${\rm{\Delta }}{i}_{\omega (x,y)}={\omega }_{(x,y)}{\rm{\Delta }}{p}_{(x,y)}{\rm{\Delta }}x{\rm{\Delta }}y$$

or formulated as density of flow *j*_(*x*,*y*)_:

6 $${\rm{\Delta }}{j}_{\alpha (x,y)}={\alpha }_{(x,y)}({\rm{\Delta }}{p}_{0}-{\rm{\Delta }}{p}_{(x,y)})$$

and

7 $${\rm{\Delta }}{j}_{\omega (x,y)}={\omega }_{(x,y)}{\rm{\Delta }}{p}_{(x,y)}$$

**Figure 11:**
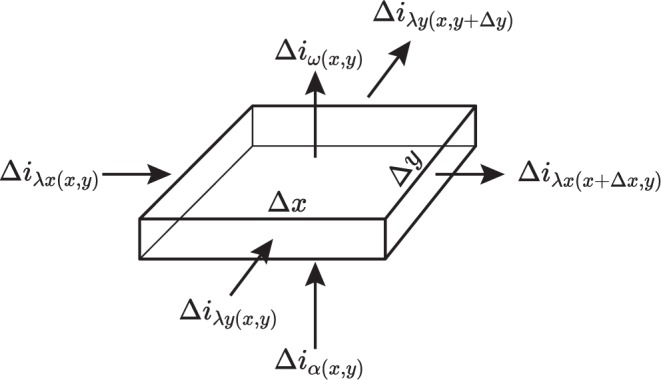
The total balance of flows to be considered in the submucous plane (8).

Also, collateral flow in the submucous plane has to be taken into consideration. *i*_*λx*(*x*,*y*)_ gives the lateral flow in axial x-, *i*_*λy*(*x*,*y*)_ in circular y-direction through the element Δ*x*Δ*y*. The total balance of flow (Fig. 11) will then be described by:

8 $${\rm{\Delta }}{i}_{\alpha (x,y)}+{\rm{\Delta }}{i}_{\lambda x(x,y)}+{\rm{\Delta }}{i}_{\lambda y(x,y)}={\rm{\Delta }}{i}_{\omega (x,y)}+{\rm{\Delta }}{i}_{\lambda x(x+{\rm{\Delta }}x,y)}+{\rm{\Delta }}{i}_{\lambda y(x,y+{\rm{\Delta }}y)}$$

Also the lateral flows can be formulated as densities of flow *j*_*λx*(*x*,*y*)_ and *j*_*λy*(*x*,*y*)_ respectively:

9 $$\begin{array}{l}{\rm{\Delta }}{i}_{\lambda x(x,y)}={j}_{\lambda x(x,y)}{\rm{\Delta }}y;\,{\rm{\Delta }}{i}_{\lambda x(x+{\rm{\Delta }}x,y)}={j}_{\lambda x(x+{\rm{\Delta }}x,y)}{\rm{\Delta }}y;\\ {\rm{\Delta }}{i}_{\lambda y(x,y)}={j}_{\lambda y(x,y)}{\rm{\Delta }}x;\,{\rm{\Delta }}{i}_{\lambda y(x,y+{\rm{\Delta }}y)}={j}_{\lambda x(x,y+{\rm{\Delta }}y)}{\rm{\Delta }}x\end{array}$$

We may now formulate the total balance of flow as:

10 $${j}_{\alpha (x,y)}{\rm{\Delta }}x{\rm{\Delta }}y+{j}_{\lambda x(x,y)}{\rm{\Delta }}y+{j}_{\lambda y(x,y)}{\rm{\Delta }}x={j}_{\omega (x,y)}{\rm{\Delta }}x{\rm{\Delta }}y+{j}_{\lambda x(x+{\rm{\Delta }}x,y)}{\rm{\Delta }}y+{j}_{\lambda y(x,y+{\rm{\Delta }}y)}{\rm{\Delta }}x$$

Also for collateral flow we will now consider local differences of pressure. The lateral resistances *λ*_*x*(*x*,*y*)_, *λ*_*y*(*x*,*y*)_ in the submucous plane are considered different in x- and y-direction and may depend on their localisation:

11 $$-\,{\rm{\Delta }}{p}_{(x+{\rm{\Delta }}x,y)}+{\rm{\Delta }}{p}_{(x,y)}={\lambda }_{x(x,y)}{j}_{\lambda x(x,y)}{\rm{\Delta }}x$$

and

12 $$-\,{\rm{\Delta }}{p}_{(x,y+{\rm{\Delta }}y)}+{\rm{\Delta }}{p}_{(x,y)}={\lambda }_{y(x,y)}{j}_{\lambda y(x,y)}{\rm{\Delta }}y$$

So (10) will develop to:

13 $${j}_{\alpha (x,y)}-{j}_{\omega (x,y)}-\frac{{j}_{\lambda x(x+{\rm{\Delta }}x,y)}-{j}_{\lambda x(x,y)}}{{\rm{\Delta }}x}-\frac{{j}_{\lambda y(x,y+{\rm{\Delta }}y)}-{j}_{\lambda y(x,y)}}{{\rm{\Delta }}y}=0$$

and considering the pressure-gradients (from (6), (7), (11), (12) as well as Δ*x* → 0, Δ*y* → 0):

14 $${\alpha }_{(x,y)}({\rm{\Delta }}{p}_{0}-{\rm{\Delta }}{p}_{(x,y)})-{\omega }_{(x,y)}{\rm{\Delta }}{p}_{(x,y)}-\frac{\partial }{\partial x}[-\,\frac{1}{{\lambda }_{x(x,y)}}\frac{\partial {p}_{(x,y)}}{\partial x}]-\frac{\partial }{\partial y}[-\,\frac{1}{{\lambda }_{y(x,y)}}\frac{\partial {p}_{(x,y)}}{\partial y}]=0$$

or rearranged and in a simplified notation:15which is the partial differential equation of the problem.

## Clinical Relevance of the Model: Results and Discussion

### Model predictions

With the given variables *ω*_(*x*,*y*)_ (conductivity of the terminal vascular bed), *α*_(*x*,*y*)_ (conductivity of the arteries perforating the muscularis propria layer) and *λ*_*x*(*x*,*y*)_, *λ*_*y*(*x*,*y*)_ (resistance to collateral flow in the submucous plane) the gradient of pressure Δ*p*_(*x*,*y*)_ can be calculated, which will constitute the *vis a tergo* for the perfusion of the terminal vascular bed at any given point (x, y) of the intestinal wall. From that we can calculate the perfusion of the terminal vascular bed *j*_*ω*(*x*,*y*)_ (terminal blood flow/area) at every location considered. The partial differential equation of intestinal blood flow has no explicit solution. With numerical methods we will however be able to estimate the influence of single variables on perfusion pressure Δ*p*_(*x*,*y*)_ and terminal blood flow *j*_*ω*(*x*,*y*)_ to the mucosa (and in the case of recurrent arteries from the submucosa also to the muscularis propria layer) as well as collateral flow in the submucous plane *j*_*λ*(*x*,*y*)_. In the following figures a Gauss-Seidel-iteration was used to show the nature of the relationship and the resulting patterns of flow^46,47^. These will be compared to the well documented site and form of human peptic ulcer in different localizations.

Terminal blood flow consists of the predominant portion of flow to the metabolically active mucosal compartment and the much smaller portion which goes to the muscle layer, as far as this is supplied recurrently from the submucous plane. Both layers are supplied by the same pressure gradient between the submucosal arterial network and the pressure level of the portal vein.

In man the topographical distribution of recurrent arteries and the varying local extent of the retrograde supply of the muscle is not established in detail. The continuous model simulates the effect of the two-dimensional submucous vascular pattern, assuming a steady distribution of mucosal and retrograde arteries. We will later discuss the effect of a variable retrograde supply of the muscle mantle.

### Gastric ulcer and related phenomena at mucosal boundaries

#### Ulcers of the proximal antrum

**Figure 12:**
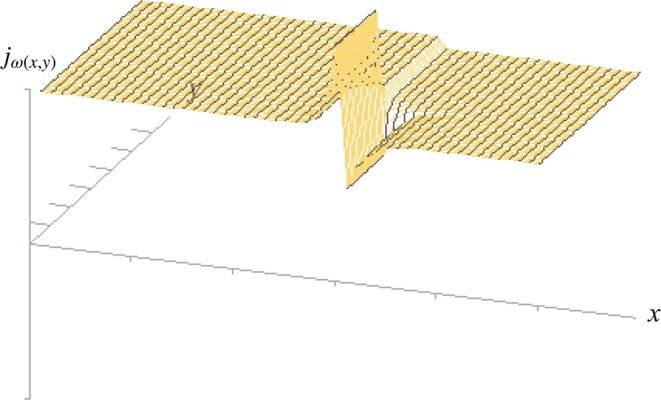
Numerical simulation for the terminal flow *j*_*ω*(*x*,*y*)_ in a 50 × 50 matrix. Initial conditions: Limited influx (*α*(*x*, *y*) = 0.1) over all segments. Conductivity of the terminal vascular bed *ω*_(*x*,*y*)_ = 5.0 in the acid-secreting corpus mucosa on the left (0 < *x* < 30) and *ω*_(*x*,*y*)_ = 1.0 in the neighbouring segments on the right (31 < *x* < 50). In the foreground (0 < *y* < 20) the lesser curvature is modelled (*λ*_*x*(*x*,*y*)_ = 3.82 × 10^−1^, *λ*_*y*(*x*,*y*)_ = 3.82 × 10^3^), in the background (21 < *y* < 50) the parietal wall (*λ*_*x*(*x*,*y*)_ = 3.82 × 10^3^, *λ*_*y*(*x*,*y*)_ = 3.82 × 10^−1^). The initial conditions are randomly chosen to illustrate the principal relationship: The lower the haemodynamic resistance in the submucosal plexus perpendicular to the mucosal border, the more marked the effect on local distribution of terminal flow will be. Numerical solution by SIMA^47^.

For the stomach the numerical simulation resembles the dissipative pattern at the mucosal boundary as it has been modelled earlier by the cellular approach. The pattern developing at the boundary between two types of mucosae with different metabolic demands shows a characteristic form: while the rise of flow to the corpus segments of the boundary will not leave any traceable pathological pattern, the loss of flow to the neighbouring antral segments will produce a loss of terminal flow which shows a steep side at the edge of the corpus mucosa and runs out shallowly in a distal direction. The result of the simulation shows the profile of blood flow _(*x*,*y*)_, which is based on the variation of Δ*p*_(*x*,*y*)_ (Fig. 12). The right part of the pattern closely resembles Aschoff’s *Reibungsform* of gastric ulcer (Fig. 2).

In clinical experience gastric ulcer is generally not correlated with acid hypersecretion. Gastric ulcers show an underlying disturbance of the motor activity of the stomach^48^. Individuals with a gastric ulcer not only show morphological signs of an alteration of the muscle wall of the antropyloric portion of the stomach^49^ but also a significant reduction of propulsive type II contractions therein^50^. Taking Aschoff’s postulate as an essential criterion for the potential role of various forms of gastritis, only the macroscopically visible phenomenon of an erosive gastritis with its maximum at the lesser curvature correlates to the site of gastric ulcers. If an ulcer is found in such a gastritic mucosa the antral gastritis generally extends caudally from the ulcer^51^. In the continuous haemodynamic model this form of an erosive gastritis would be the least amount of circulatory injury of the mucosa to be expected. It will appear as the outermost ring of macroscopically detectable tissue damage asymmetrically surrounding the eccentric ulcer cockade.

The form and site of antral ulcers (Fig. 2) correspond to the predictions of the differential haemodynamic model (Fig. 12). To further test the predictive power of the model we will compare the outcome of the simulation to rarer peptic lesions which eventually show up at anomalous locations in the stomach.

#### Ulcers of the cardia

**Figure 13:**
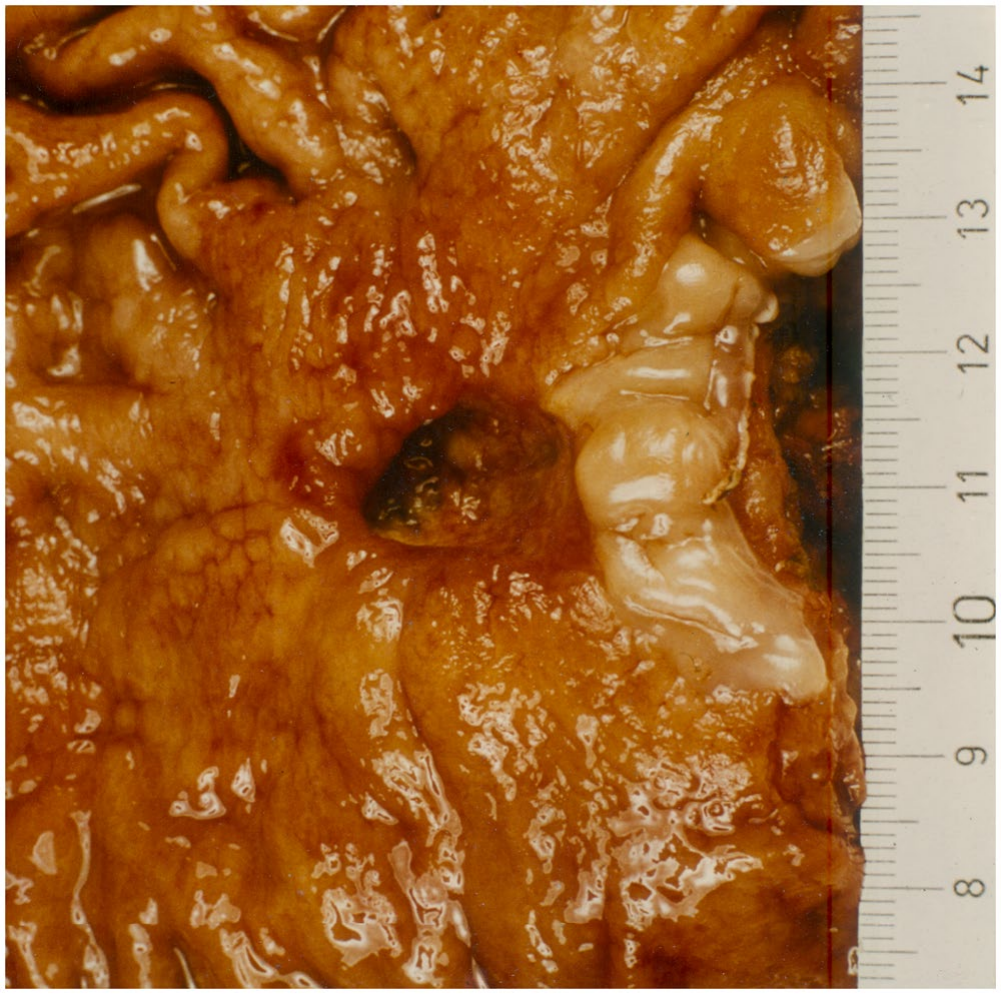
Operative specimen with a benign ulcer of the cardia. These rare lesions can be found on the cranial edge of the corpus mucosa. The squamous epithelium of the oesophagus is visible in a whitish colour at the right. In contrast to Aschoff’s assumption of a secondary mechanical alteration of a cylindrical necrosis the ulcer at the cardia shows a funnel pointing in the opposite direction (against the peristaltic propulsion): a steep and overhanging edge at the corpus-side of the lesion, running shallowly out in a proximal direction towards the oesophagus. It mirrors the form of the typical gastric ulcer at the antrum/corpus-boundary (Fig. 2) and is correctly predicted by the differential haemodynamic model (Fig. 12).

**Figure 14:**
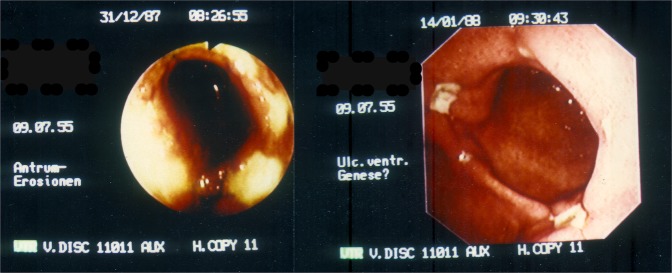
Endoscopic view of an acute linear lesion in the distal antrum. The picture has been taken during an emergency situation but the necrotic mucosa can be clearly seen extending clay-coloured all around the circumference in a circular fashion (left). Two weeks after medical inhibition of acid secretion the damaged mucosa is largely restored leaving only small rest ulcers behind which are situated in a cross-section around the circumference (right). Endoscopic observation: HJ Dittler.

Ulcers at the cranial (opposite) edge of the acid-secreting mucosa are rare phenomena. At the cardia, the muscle layer is less pronounced than it is in the distal stomach, so the arterial influx to the submucous plane will be less compromised than further distally at the antrum/corpus-boundary. Oi described one ulcer of the cardia at the proximal edge of the corpus mucosa in 500 cases of gastric ulcer^17^. The form of such a rare ulcer of the cardia (Fig. 13) mirrors the symmetry of the typical antral ulcer (Fig. 2). The form of this ulcer falsifies Aschoff’s attempted explanation of a secondary mechanical alteration of the ulcer morphology. It shows a laterally reversed image, as compared to the form of the typical gastric ulcer at the antrum/corpus-boundary. Importantly, the morphology of the ulcer at the cardia is correctly predicted by the continuous model Fig. 14.

Ulcers at the cardia which occur in a hiatal hernia have been described as a separate entity due to their intrathoracic localisation. Oi named his above mentioned example of an ulcer at the cardial margin as an “oesophageal” ulcer^17^. From a pathophysiological point of view, these ulcers should be regarded as ulcers of the cardia, wherever this structure is located, as long as they occur at the cranial boundary of the parietal cell mass.

#### Linear ulcers

Round ulcers of both shoulders of the parietal cell mass are consistent with the predictions of the differential approach. The course of the vessels in the submucous plane is sufficient to determine their site and form.

In regions where such characteristic circumferential differences in the submucosal arterial pattern cannot be observed the effect should no longer be limited to only a part of the circumference. Such conditions exist in the distal antrum and proximally in the tubular oesophagus^37,41^. If in younger persons the boundary of the acid-secreting mucosa reaches far into the distal antrum, the circumferential anisotropy of the submucous plexus as we find it in the corpus and proximal antrum is no longer pronounced (left of Fig. 9). When the submucous arteries show no preferential direction, no differences in the longitudinal resistance *λ*_*x*(*x*,*y*)_ of the submucous compartment are to be expected around the circumference. A simulation of MBF using the PDE of intestinal blood flow at such a distal part of the antrum will produce a circular pattern at the mucosal boundary. The corresponding pathological pattern expected will be be a circular wound. Such lesions are well known in human pathology (Fig. 14). They have been called linear ulcers^52–54^. Clinically such linear ulcers have only been found at far distal sites in the antrum and in combination with an antral stenosis.

#### Peptic stricture of the tubular oesophagus

The midoesophageal peptic stricture, which develops inside the tubular oesophagus, is not in the scope of the model as our reduction (i) does not apply: the columnar epithelium which forms the mucosa distal to the stricture does not contain any parietal cells^55^. Nonetheless, the stenosis does develop at the borderline between two different epithelial structures which differ fundamentally in their metabolic demands. While the oesophagus proximal to the stenosis is lined by a multilayered squamous epithelium without any secretory activity, distally we find a metabolically active columnar epithelium, named Barrett’s mucosa^56,57^. With axial submucous arteries running around the circumference here again we find the anatomical conditions for the development of a circular, non-occlusive infarct. From the surgical experience any complete circular necrosis of the lining surface epithelium in a small hollow structure like the oesophagus would necessarily lead to a fibrous stenosis at this juncture.

A midoesophageal stricture is only found in the combination of an endobrachyoesophagus (Barret’s oesophagus) and an insufficiency of the cardia. While physiologically the stomach is closed towards the oesophageal lumen, in this situation stomach and oesophagus form one situational continuous cavity - any elevation of intragastric pressure will continue into the oesophageal lumen. The stricture develops in only a small percentage of patients who show this combination. Clinical observations point to the third precondition for the development of a midoesophageal stricture, that of a disturbed motor function of the antrum: surgically these lesions have been successfully treated by a limited antral resection alone. Wangensteen had introduced this simple procedure (removal of the pylorus and the distal antrum) in 1949 - it leads to a spectacular healing of the circular stenosis within weeks by an operation far distal to the lesion^58,59^.

The continuous haemodynamic model allows a simple coherent explanation of the observed effect: any limited resection of the pyloric antrum will prohibit longer lasting episodes of intraluminal pressure in the situationally now continuous cavity of stomach and oesophagus. This will necessarily remove the cause for any localised disturbance of blood flow, even when the mucosal borderline is placed in the tubular oesophagus.

#### Limitations of the borderline approach

In all of the above mentioned cases the anatomical picture of a circumscribed necrosis can be explained functionally by the energy requirements of secretion under the condition of a limited arterial influx alone. Neither an occluded vessel nor the presence of unbuffered intraluminal acid (iv) is relevant to understand the morphology of functional necrosis of the stomach in a complex haemodynamic network. Gastric ulcer will be found at that part of the circumference where the anatomical course of the submucous arteries promotes a redistribution of blood flow perpendicular towards the mucosa with the higher energy requirements. This will only occur when the influx to the submucous plexus is limited by the muscle mantle. From a pathophysiological point of view all the lesions mentioned above should be classified together as borderline lesions. Circumferentially they will either be limited to places where the structure of the submucous plexus provides an anisometry, as Waldeyer’s *Magenstrasse*, or they will be circular when the parietal cell mass extends to the outlying or neighbouring parts of the organ, where an isotropic pattern of submucous arteries has prevailed around the circumference. In clinical experience, borderline ulcers generally share a disturbed motor function (ii) while hypersecretion (iii) is not an essential factor in their causal pathogenesis^48–50^. Generally the ulcer is typically a solitary phenomenon - the amount of acid produced does not correlate with the amount of tissue damage but with the site of the ulcer. Historically also Schwarz’s famous dictum “no acid, no ulcer” has been overstated as a pathophysiological principle. While it represents a correct observation for duodenal ulcer it should be carefully restated more precisely for gastric ulcer as “no acid *secretion*, no ulcer”. Certainly, such a less misleading formulation would still neglect the fundamental sine qua non of wall stress phenomena in the development of gastric lesions. But first and foremost the dictum has never provided an answer to Aschoff’s fundamental problem. Historically, the close correlation of a gastritis and the occurrence of ulcer had early led to the assumption of a causal interrelationship between these two entities^60,61^. It is obvious that such a proposed progression from a gastritis to an ulcer faces the same principal inconsistency as any surface theory. The distributive pattern of histologically distinct types of a gastritis in an ulcer-bearing mucosa does generally not match the morphological characteristics of the ulcer^62^. Using the model reductions we will be able to show that any inflammation is not a necessary condition in the formal pathogenesis of a deep-walled necrosis of the intestinal wall.

### Duodenal Ulcer and related non-borderline phenomena

As far as the aetiology and clinical aspects of gastric and duodenal ulcers are concerned, they seem to present two separate entities: in gastric ulcer none of Koch’s postulates is satisfied for H.pylori. It is neither detectable in all instances of the lesion nor can gastric ulcer be produced by the installation of the bacterium. For duodenal ulcer, Koch’s first postulate is largely satisfied while the second is not. Thus the presence of H. pylori does not necessarily lead to a duodenal ulcer. From the morphological perspective, however, identically formed and sited duodenal ulcers can be found in rarer conditions such as gastrin-producing tumours as well. Duodenal ulcers correlate to a sustained secretion of hydrochloric acid under fasted conditions. Helicobacter pylori inhibits the duodenal feedback mechanism that prevents secretion of HCl in a fasted stomach. It has been identified as the single most important etiologic factor for acid hypersecretion^2,3^.

Oi had stressed the point that, not only gastric but also duodenal ulcers appear adjacent to a transitional zone (between pyloric glands and the duodenal mucosa). With the metabolic demand of the proximal duodenum exceeding gastric MBF in the acid-secreting areas, a haemodynamic mechanism that mirrors the mechanism at the antrum/corpus-boundary seems to be an attractive hypothesis at first glance. A careful look at Oi’s findings, however, reveals that he had defined “adjacent” as closer than 2 *cm* to the histological border. For duodenal ulcer Oi’s conclusion could turn out to be a circular argument - duodenal ulcer only occurs at the duodenal bulb with its dual mesenteric supply ending 3–4 *cm* distal to the pylorus. The mean distance between the duodenal ulcers Oi described was found to be only 0.42 *cm*. In the Japanese study 44 out of 136 duodenal ulcers were located at the border zone, while 91 were located in the duodenal gland area distal to the histological border. While the boundary between antral and duodenal mucosa is generally located near the pylorus muscle, variations do exist. Duodenal mucosa could be found up to 2 *cm* proximal to the pylorus. Brunner’s glands (a typical feature of the duodenum) have also been detected in the distal stomach. Antral mucosa can also extend distal to the pylorus^17,63,64^. It has also been speculated, that gastric metaplasia at the edge of duodenal ulcers may be a common cause for their emergence^65,66^.

A simple steal mechanism as we described it at gastric borderlines would, however, only work in an area with limited transmural influx as we may find it over the pylorus muscle or in cases where the duodenal mucosa extends into the distal antrum with its heavy muscle mantle but never in the duodenal bulb itself. The existence of dysplastic mucosal islets constitutes an interesting hypothesis in the rare cases of gastric ulcers not occurring at borderlines but it will not be useful to describe the typical morphology of duodenal ulcer.

We will continue to strictly focus on the formal mechanism of ulcer formation. Aschoff’s postulate brings another argument into the old dispute on the nature of peptic ulcer: Gastric ulcers of the proximal antrum exhibit a sharp border towards the parietal cell mass and run out shallowly away from the acid-secreting mucosa. In contrast, ulcers developing inside the duodenal mucosa display a rotational symmetry with acute borders at all sides. For duodenal ulcer we will have to show the conditions for the possible development of a supply/demand conflict under the local condition of a homogenous mucosa as well as the morphological outcome of such a condition.

Neither the discrete haemodynamic model nor the cellular automaton have been able to produce the typical aspect of a duodenal ulcer within a continuous mucosa and away from any mucosal boundaries.

From the morphological point of view duodenal ulcer is found at the anterior and/or posterior aspect of the duodenal bulb in-between the supplying muscle-piercing arteries in a homogenous mucosa devoid of mucosal boundary conditions. Haemodynamically, both in terms of embryological development and the pattern of its submucosal arteries, the duodenal bulb is a part of the foregut. Compared to the stomach the submucous pattern of the duodenal bulb is rather uniform. The only major source of anisotropy in the submucosal network around the circumference of the bulb is its bilateral mesenteric blood supply. This anatomical peculiarity typically ends about 3–4 *cm* distal to the pylorus. Duodenal ulcer is a phenomenon which is strictly limited to this short segment. In areas where such a direct transmuscular influx by perforating arteries is not provided, the arterial supply of the metabolically active mucosa will depend on submucosal collaterals only. Can the characteristic pattern of a supply/demand conflict developing in such a watershed area be simulated by use of the considered variables alone?

(14) shows, that any pattern of 0 > Δ*p*_(*x*,*y*)_ > Δ*p*_0_ in a homogenuous mucosa can be produced with a rise in mucosal conductivity (*ω*↗) or a limitation of transmural influx (*α*↘) as well. Expressed in a simple term: the pattern of any supply/demand conflict in a given vascular structure (*λ*_*x*(*x*,*y*)_, *λ*_*y*(*x*,*y*)_) can either arise through a limitation of blood supply or alternatively by an excessive rise in demand as well. Pathologia physiologiam illustrat: when similar morphological effects can be produced by either elevating wall stress or boosting mucosal blood flow we should have a look for both conditions in human pathology.

#### Wall stress effects

The differential model describes blood flow not only in the walls of the stomach but in any structure which has the principal layout of the intestinal wall: perforating arteries piercing through the muscle layer on their way towards the mucosa forming a collateral system in the submucosa.

*Singular ulcer of the rectum*: Far distal from any structure which has contact with hydrochloric acid we do find another round ulcer in the lower rectum of man. This singular ulcer of the rectum occurs under the conditions of a limited influx to a structure which, here again, has a bilateral arterial supply and a marked arterial collateral system in the submucosa. An incomplete rectal prolapse is always associated with these lesions. The anterior wall of the rectum forms the intussusceptum of the beginning prolapse. It is exposed to the constant contraction of the sphincter muscle and the pelvic floor which may cause a long lasting limitation of influx. The singular ulcer of the rectum is strictly confined to the affected anterior wall of the rectum and it closely resembles the rotationally symmetric shape of duodenal ulcer^67,68^.

**Figure 15:**
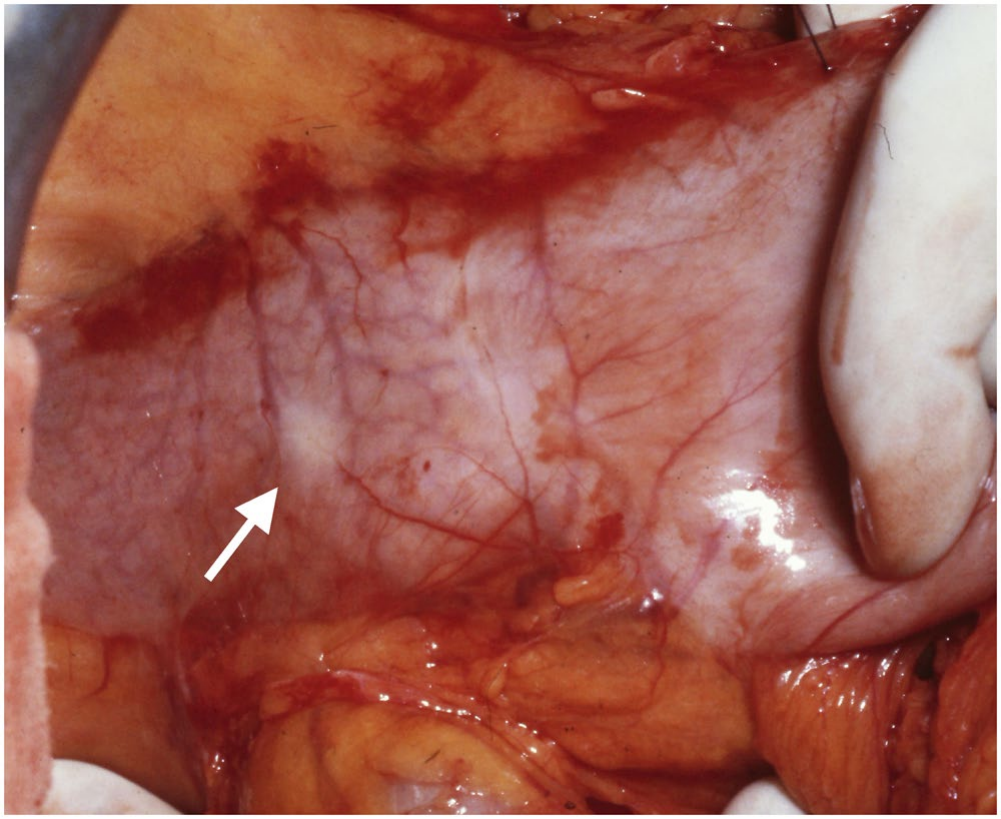
Mayo’s anemic spot demonstrated at operation in a view of the anterior wall of the antrum and the proximal duodenum. Dragging the antrum away from the fixed duodenum into the wound, a pale spot will appear at the anterior aspect of the bulb (left). It will vanish completely as soon as the tension is released. It develops at the typical site of duodenal ulcer and may be confused with it. However it does not show the sharp delineation of a duodenal ulcer. A second whitish structure running transversely can be seen directly over the pyloric muscle (centre), the most prominent muscular structure in the foregut.

*Mayo’s anemic spot*: In the human duodenum such extreme and long-lasting elevations of wall stress do not occur during a digestive cycle. Nevertheless, the requirement to expose more deeply located structures during upper abdominal surgery means that the effects of wall stress can be observed at operation. In 1908 William Mayo erroneously resected a stomach on the assumption to encounter a duodenal ulcer, which he did not find in the resected specimen after operation^69^. He observed a radially symmetric ischemic spot at the anterior aspect of the duodenal bulb, which he confused with a duodenal ulcer. However, the phenomenon he observed was of temporary and reversible nature. Mayo’s anemic spot is now a well known phenomenon among surgeons. It shows up at the typical site of duodenal ulcer though it does not display the acute margins of an ulcer. It only develops when the duodenum is subject to wall stress between the (fixating) hepatoduodenal ligament and the pull exerted by the hand of the observer. It vanishes as soon as the grip of the surgeon’s hand is released (Fig. 15).

Mayo’s anemic spot is reproducible at operation but it is not a consistent phenomenon. As a clearly visible circular spot it is only found in about a quarter of attempted observations. R.M. Kirk described a less marked pale area at the anterior duodenal wall in 89% of individuals at operation^70^. Clearly the anatomical peculiarity which is needed to produce an uneven distribution of blood flow to the outer parts of the duodenal wall by traction varies between individuals. Accordingly a morphological predisposition appears essential to this expression of a limited flow phenomenon to the duodenal bulb under the influence of wall-stress (*α*↘). A well defined anemic spot also reveals that the recurrent arterial supply to this part of the duodenal wall reaches from the submucous plane as far as the subserosal level in order to be clearly visible to the naked eye, at least in the 25% to 30% of cases where the phenomenon can be observed.

#### Duodenal ulcer

**Figure 16:**
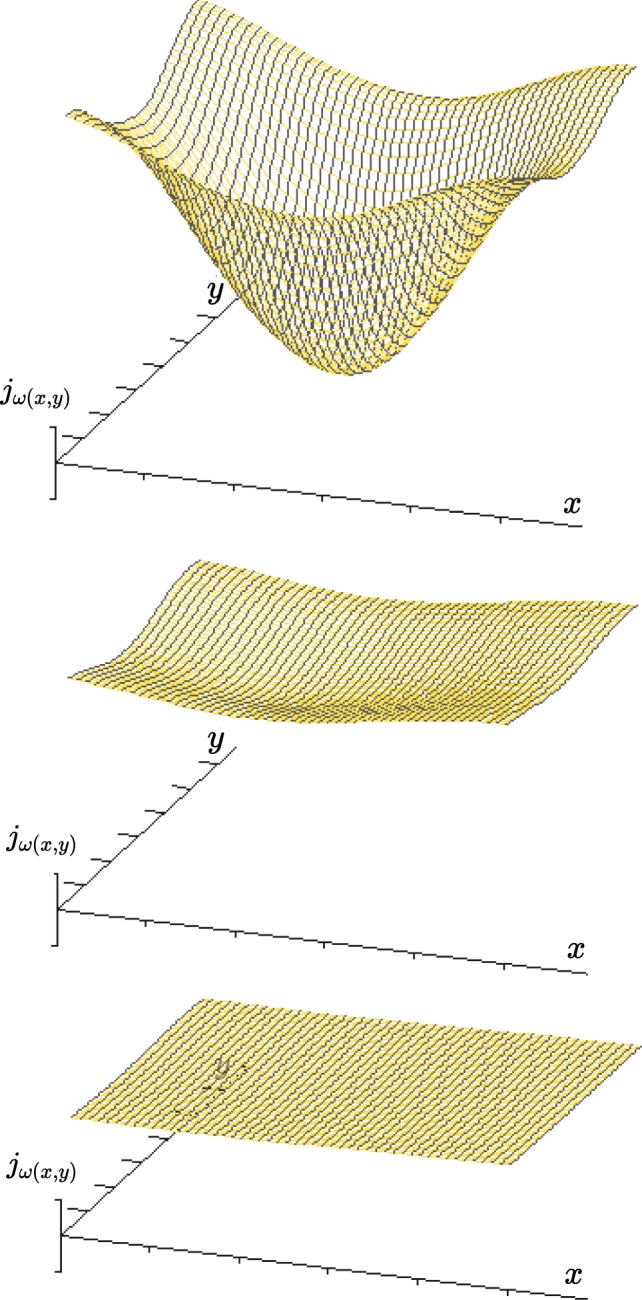
Simulation of the influence of a rising mucosal conductivity (*ω*↗) on blood flow in the terminal vascular bed: Terminal blood flow *j*_*ω*(*x*,*y*)_ in a rectangular limited watershed area of the duodenal wall, which is only collaterally perfused. Considering the localisation of Mayo’s anemic spot such areas with a strictly retrograde flow to the muscle mantle do exist to some extent at the anterior and posterior aspect of the duodenal bulb. In the central 30 × 30 area, which has no direct transmural influx but is supplied by submucosal arteries only, mucosal flow is maintained by collateral flow alone. When local blood flow rises, adapting to an increasing metabolic demand, the effect will be more marked - in strictly collaterally supplied areas a central dip of MBF will develop. Disregarding the initial form of the collaterally supplied area it will produce a nearly circular profile at the bottom. The same effect can be accomplished by increasing wall stress (*α*↘). In both cases collateral flow will exclude any discontinuity of terminal flow, as long as the mucosa is vital: the effect will not be limited sharply. Initial conditions: in the 50 × 50 matrix considered there is no direct transmural influx to the central 30 × 30 square (*α*_(*x*,*y*)_: = 0.001), while the surrounding (10, 10) 〈(*x*, *y*)〉 (41, 41) has a free influx (*α*_(*x*,*y*)_: = 1). A uniform criss-cross-pattern of submucous arteries is assumed: (1, 1) < (*x*, *y*) < (50, 50) → *λ*_*x*(*x*,*y*)_ = *λ*_*y*(*x*,*y*)_ = 3.82 × 10^−1^. Mucosal metabolic demand and conductivity *ω*_(*x*,*y*)_ will rise exponentially from the bottom to the top: *ω*_(*x*,*y*)_: = 1 (*bottom*), *ω*_(*x*,*y*)_: = 10 (*centre*), *ω*_(*x*,*y*)_: = 100 (*top*). Numerical solution by SIMA^47^.

A very similar effect on blood flow can be simulated by excessively increasing the metabolic demand of the duodenal mucosa as a result of acid attack (*ω*↗). In the case of an intraluminal load of unbuffered acid, mucosal blood flow will rise to values which far exceed blood flow in the acid-secreting areas. In an underlying anisotropic structure, any augmentation of mucosal conductivity due to an extended metabolic demand cannot be answered uniformly by local blood flow even when the mucosal metabolic response to such a stimulus is uniform. Such a watershed area with mainly collateral arterial supply to the mucous membrane can be found at the duodenal bulb in-between the transmural arterial inflows from both mesenteries at the site where Mayo’s anemic spot can be observed. The resulting blood flow may be insufficient to meet the metabolic demands necessary to defend the mucosa against acid attack. The developing pattern of mucosal flow will have a circular aspect in the centre but, like Mayo’s anemic spot, will not be sharply delimited as long as the mucosa is vital. To demonstrate that outcome we have deliberately chosen a quadratic area with strictly collateral supply for the example given in Fig. 16.

**Figure 17:**
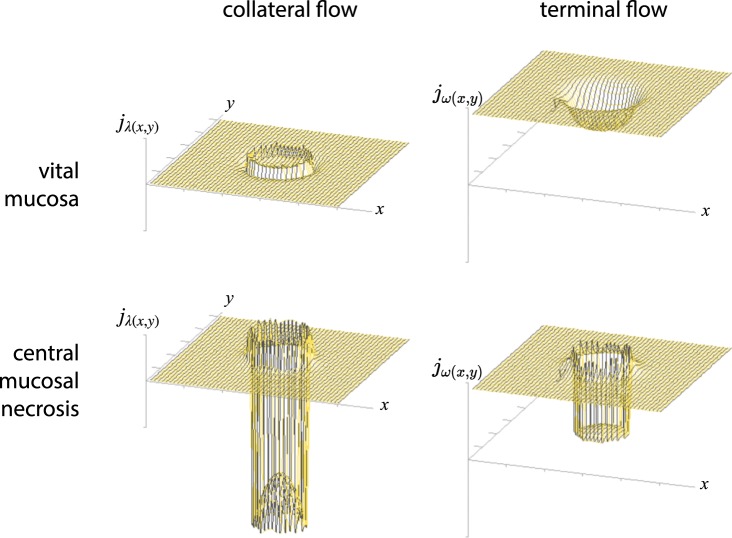
The influence of mucosal viability on local distribution of flow to the duodenal wall. A circular watershed area (ø_*wall*_ = 18) with predominantly collateral supply is considered in the centre of the 50 × 50 matrix (*top*). This central area has a limited direct transmuscular influx (*α*_*centre*_: = 0.05). The surrounding area is considered to have a direct access to transmuscular supply (*α*_*surround*_: = 0.1). In the submucosa arteries are evenly distributed in both directions: *λ*_*x*(*x*,*y*)_ = *λ*_*y*(*x*,*y*)_ = 3.82 × 10^−1^. Mucosal metabolic demand and conductivity *ω*_(*x*,*y*)_ = 10 in the vital mucosa (*top*). When the resulting blood flow falls below a threshold level, which is assumed the minimum necessary to defend the mucous membrane against acid attack, the mucosa will become necrotic. The remaining conductivity of the centre will now be given by the remaining retrograde flow to the surviving parts of the muscularis propria layer: we assume the vascular resistance of dead tissue to rise extremely and set the remaining conductivity of the centre *ω*_*centre*_: = 0.05 (*bottom*). As long as the mucosa is viable, collateral flow will be directed towards this part of the wall, *j*_*λcentre*_ > 0 (*top left*). For blood flow in the terminal vascular bed this will produce a continuous drop in flow to areas with a prevailing collateral supply (*top right*). As soon as mucosal viability breaks down at the tip of the dip (ø_*wall*_ = 15), collateral flow will dissipate away from this area *j*_*λ*(*x*,*y*)_ < 0 (*bottom left*). Only now will local blood flow to the centre of this area totally fail (*bottom right*). Due to the reversal of collateral flow a contrast enhancement will develop at the borders of the necrotic area: the pattern resembles that in Fig. 12 in a circular fashion with the negative values adding in the centre. The local redistribution of collateral flow *j*_*λcentre*_ is again directed away from the necrotic centre towards the surrounding area, which is still vital. By the structure of the network alone the effect on local distribution of flow will produce a self limiting effect, which is in the final analysis only based on Kirchhoff’s laws. Numerical solution by SIMA^47^.

If this intermediate state lasts long enough, a perfusion breakdown of corresponding muscular layers which are supplied by retrograde arteries alone should be observable. Such muscular necroses underneath a viable mucosa with an intact basal membrane have been described by Victor Hoffmann opposite to acute duodenal ulcers^71^. In his resection specimens Hoffmann systematically examined the opposing wall of the duodenal bulb at the site where corresponding “*kissing*” ulcers develop. His disturbing histopathological findings could not be explained at the time of their description and have been neglected ever since. If, alternatively, the resulting mucosal blood flow to the centre of the dip becomes insufficient to meet the metabolic demands necessary to defend against a prolonged acid attack this will dramatically change the spatial profile of blood flow to the margins of the area affected (Fig. 17). Such a breakdown of mucosal circulation will lead to a further redistribution of flow. A dissipative pattern similar to the one at mucosal boundaries will develop around the circumference of the ulcer. With a mucosal necrosis in the centre we again face two types of mucosae which will differ in their conductivity. With the conductivity of the necrotic mucosa approaching zero, the remaining conductivity of the terminal vascular bed *ω*_(*x*,*y*)_ will break down to the remaining conductivity of the vital part of the muscle layer, which is supplied from the submucous plexus alone. The developing pattern will produce a contrast enhancement of local flow between viable and necrotic parts of the wall. The pattern will show a well defined margin - producing a rotationally symmetrical lesion which is self-limiting in size and sharply demarcated from a surrounding area with nearly unaffected blood flow (Fig. 17).

**Figure 18:**
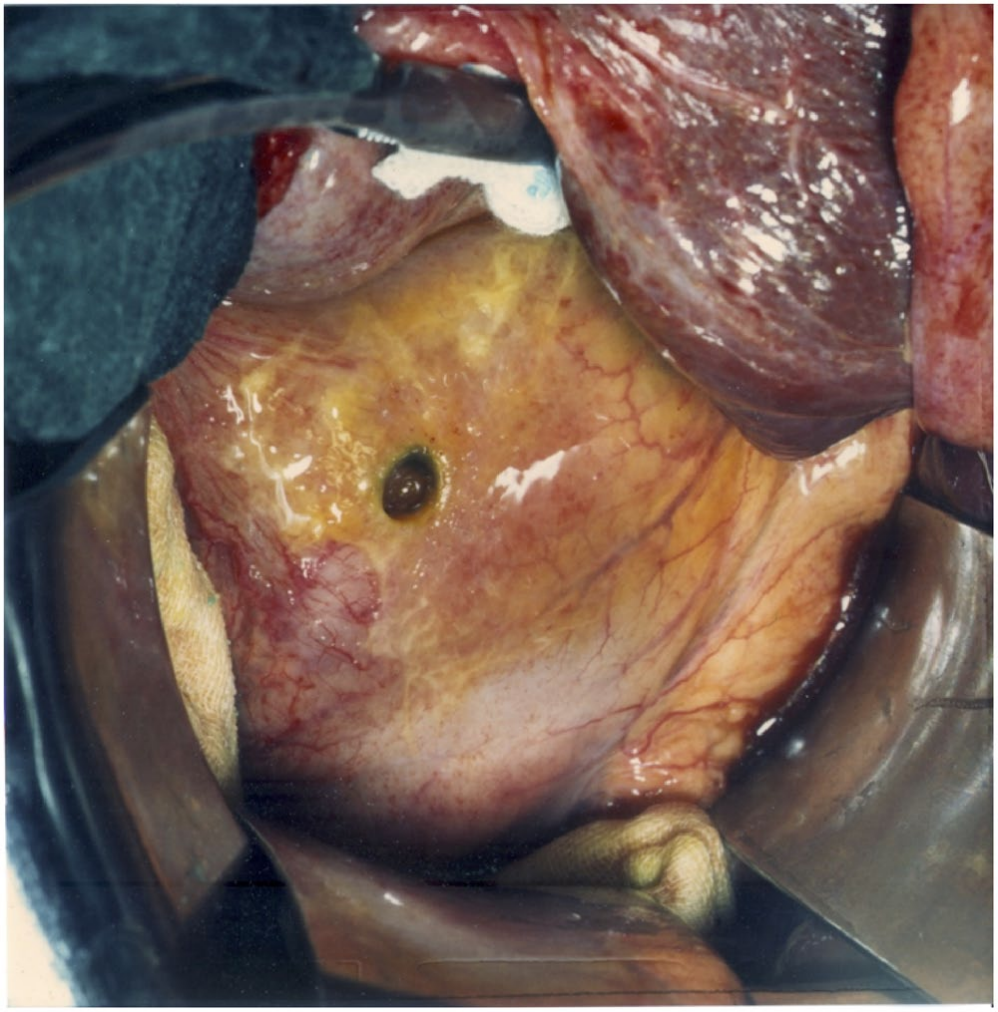
View at a freshly perforated duodenal ulcer at operation. The characteristic form of this full wall necrosis develops at the anterior surface in-between the arterial influx from both mesenteries. The direct view from the serosal side shows the radial symmetry of the transmural defect with acute margins to all sides of the vital surrounding tissue. Macroscopically and microscopically this typical finding appears to be punched out from a grossly intact duodenal wall.

Only now, ostensibly by a contrasting enhancement in blood flow to the surrounding, will a well defined defect in blood flow from the submucosal plane be formed. The margins of the original deficit will become stabilised by a reversal of flow in the submucous plane away from the initial defect and towards the surrounding. What seems to look like a defensive mechanism to prevent further damage of the surrounding is an inherent property of the structure of the submucous mesh. In the final analysis the form of duodenal ulcer (Fig. 18) confirms the validity of Kirchhoff’s laws in a complex haemodynamic network.

Such a sharply limited defect of local blood flow will produce a necrotic cylinder, the depth of which only depends on the distribution pattern of recurrent arteries to the muscle layer. Its shape will depend on the dissipative pattern (Fig. 17, *bottom*), which develops secondary to the initial mucosal necrosis. The clinical fate of the ulcer (*to perforate or not to perforate*) will finally be predetermined by the local extension of recurrent arteries originating at the submucous plexus and supplying the muscle mantle. If they supply the full thickness of the wall, the anatomical conditions for a full wall necrosis are met. Not a single observation but our language alone implies that “*perforation*” or “*penetration*” are the consequence of forces which show a preferential direction from the lumen to the outer walls of the intestine. Perforation is an acute process, which is not due to the prolonged action of a surface irritant alone, but depends primarily on the pattern of retrograde muscular supply in an area which predominantly depends on submucous collateral flow.

To this day our anatomical knowledge of the variation in the topographical distribution patterns of recurrent arteries in the duodenal bulb and also the stomach is far from being systematic. Anatomists have mostly focused on the mucosal supply and, in the course of injection studies, often dissected the muscle mantle away. Nonetheless clinical observations should be consistent with the theoretical model.

The distribution pattern of submucosal arteries is generally similar on the anterior and the posterior surface of the duodenal bulb. For any pathological condition which can be found at the anterior wall there should be an equivalent on the posterior wall of the duodenal bulb. It is not so unusual for duodenal ulcers to be expressed on both walls simultaneously - *kissing ulcers* is the name assigned to this pathological condition. Again, the name seems to imply a surface mechanism and neglects the finding of deep-wall necroses of the intestinal wall opposing an acute duodenal ulcer, as they have been first described by Victor Hoffmann - his findings alone falsify every surface theory of ulcer formation^71^.

Ulcers at the anterior or posterior wall usually differ in their size and their typical clinical outcomes although the vascular morphology of the duodenal bulb on both sides typically does not. The anterior wall of the bulb directly faces the abdominal cavity. Any perforation here will immediately produce the potentially fatal condition of a peritonitis, which will hardly go unnoticed. Perforated ulcers at the anterior aspect of the duodenal bulb are usually smaller than chronic duodenal ulcers of the posterior surface. Acute perforations at the anterior circumference often are not bigger than a pinhead but they cause a severe condition. At the posterior wall such small lesions are usually noticed only in the event of a massive haemorrhage. As long as massive bleeding does not occur, pain is the only symptom of ulcers on the posterior wall. Here the process will not be limited in size by any fatal condition. As long as the conditions for a circumscribed redistribution prevail the process can start over and over again, although the maximum diameter of duodenal ulcers is limited^72^. Eventually also a full thickness necrosis of the posterior wall will lead to fatal consequences. Bigger ulcers of the posterior wall finally expose the surface of larger arteries at the ventral aspect of the pancreas to the influence of unbuffered hydrochloric acid - massive haemorrhage is the typical risk of duodenal ulcers at this site.

Generally the extent of the loss of tissue substance in round ulcers varies wherever they are observed: while benign giant ulcers are well known in the antrum^73^ they are limited in their maximal size to the area in-between the muscle perforating sources of the duodenal bulb. While their maximum extent is only defined by the meshes of the submucous network and the area which shows a predominantly collateral supply of these parts of the wall, the minimal size of any supply/demand conflict will be defined by the necrotic cylinder surrounding a single central artery. Such minimal lesions have been observed early as a cause for gastrointestinal haemorrhage. They were first described by Georges Dieulafoy in^74^. Consistently, the endoscopic observer today will notice the bleeding stump of the central artery in the bottom of these “*simple exulcerations*”. Historically this has led to the postulate of an underlying vascular disorder. However, such small necroses around a central artery are in the scope of the partial differential model without this additional assumption. Dieulafoy’s lesions have been described not only in the upper gastrointestinal tract but also in the rectum^68,75^.

## Conclusion

Since the days of Rudolf Virchow^4^ the problem of localizing factors in the pathogenesis of a singular human gastrointestinal ulcer has been tackled repeatedly. In the past 30 years the focus of interest has completely shifted to the aetiology of acid hypersecretion and its possible implications. Formal aspects of ulcer development have been eclipsed by these developments. Ludwig Aschoff’s initially quoted postulate^1^ stems from a lecture in 1912. It addresses the formal pathogenesis of human ulcer. It is clear that Aschoff’s fundamental question did not find the attention it well deserves. The morphological basis for the model proposed in this paper has been known from around Aschoff’s time or shortly thereafter. Aschoff’s problem can be solved in a simple fashion by a differential approach to intestinal blood flow.

The differential haemodynamic model of the intestinal wall is capable of explaining form and site of gastric and duodenal ulcers in man. The historical antagonism between surface and vascular theories of ulcer pathogenesis becomes pointless as we widen our scope and examine in detail the structure of the arterial submucous plexus. The results of the model presented here do not negate our knowledge about the aetiology of acid hypersecretion in the development of duodenal lesions. As far as the formal pathogenesis of duodenal and gastric ulcers is concerned there is a clearcut difference between these two lesions. The morphology of gastric ulcer can be understood based upon the energy requirements of acid secretion and the limited arterial influx through a contracted muscle mantle in the given vascular supply of the gastric wall. The development of duodenal ulcer, however, requires an extreme rise in blood flow, as it will be provoked by a prolonged direct contact of unbuffered acid with the duodenal mucosa. Generally, in an anisotropic structure such as the human arterial submucous plexus, the metabolic demands of the mucosa will not be met uniformly. The resulting pattern will depend on the individual anatomical distribution of vessels in the wall of the stomach and duodenum.

Using Aschoff’s postulate as Occam’s razor it becomes obvious that a progression from a gastritis to an ulcer faces the same principal inconsistency as any surface theory, namely, it does not provide any explanation as to the appearance of a spatially limited and circumscribed ulcer against a background of generalized inflammation. The proposed sequence of gastritis to ulcer is merely based on the correlation of these two entities. It has never lead to any formulation of a consistent theory of the formal pathogenesis of human ulcer that accounts for morphology and localization. Areas of increased acid susceptability of the mucosa are not in the scope of the model but have to be considered as independent phenomena. Of particular note, a gastritis of whatever form is not among the variables (i–iv) required to produce the characteristic pattern of a functional infarct of the intestinal wall. Importantly, also a Helicobacter-associated gastritis constitutes neither a sufficient nor a necessary condition in the development of an ulcer but an epiphenomenon of colonization.

In anatomical research there is still a gap between the topographical distribution of named vessels in man^76^ and the spatial microvascular patterns demonstrated using SEM-techniques on human specimens^27,77,78^. The course and diameter of intramural gastric arteries in the sub-millimeter-range^37^ will be essential for any individual simulation of the complex three-dimensional network. With a continuous haemodynamic approach we do have the instrument, although we so far lack a topographical approach to the distribution pattern of recurrent arteries in the human stomach and duodenum. The variance in the expression of Mayo’s anemic spot strongly suggests that there are systematic differences in their individual distribution and retrograde range. Only if we understand the individual variation of retrograde supply to the muscle mantle will it be possible to predict which ulcers perforate and which don’t.

In principle it will never be possible to extrapolate backwards from an established ulcer to the underlying vascular pattern in the submucosa. In chronic ulcers this is prevented due to the loss of tissue substance and the process of scarring. However, we should be able to use the partial differential equation of intestinal blood flow to run a complete individual simulation in man without violating the arrow of causality. Nevertheless, only if the open anatomical questions are answered will we be able to use finite methods to prospectively decide on vascular predispositions for the expected depth of an ulcer in single individuals.

If we look at what has been known for a century about the two-dimensional patterns of the arterial submucous plexus in the human stomach and duodenum, a differential haemodynamic model is capable of predicting the possibility of localised disturbances of blood flow of a specific pattern and localisation. Both these aspects of blood flow match up with our current understanding of ’peptic’ ulcer and related lesions. Thus, without the need for any further assumptions, a functional non-occlusive infarct based upon a supply/demand conflict presents a consistent explanation for the formal pathogenesis of a circumscribed ischaemic necrosis in the stomach or duodenum. A local redistribution of blood flow at the submucous level encompasses both the clinical and morphological findings with regard to singular ulcers in the human gastrointestinal tract.
